# Silage Fermentation on Sweet Sorghum Whole Plant for Fen-Flavor Baijiu

**DOI:** 10.3390/foods10071477

**Published:** 2021-06-25

**Authors:** Hongshen Li, Xinglin Han, Hongrui Liu, Jianqin Hao, Wei Jiang, Shizhong Li

**Affiliations:** 1 Institute of New Energy Technology, Tsinghua University, Beijing 100084, China; li-hs17@tsinghua.org.cn (H.L.); hongruiliu@mail.tsinghua.edu.cn (H.L.); 2 Beijing Engineering Research Center for Biofuels, Beijing 100084, China; 3 China National Research Institute of Food and Fermentation Industries, Beijing 100015, China; hanian2009@163.com (X.H.); haojianqin@163.com (J.H.); jiangweigyig@163.com (W.J.); 4 Beijing Laboratory for Food Quality and Safety, Beijing Technology & Business University, Beijing 100048, China

**Keywords:** Baijiu (Chinese liquor), *Saccharomyces cerevisiae*, sweet sorghum, silage fermentation, volatile flavor

## Abstract

The technology for producing bioethanol from sweet sorghum stalks by solid-state fermentation has developed rapidly in recent years, and has many similarities with traditional Chinese liquor production. However, the product from sweet sorghum stalks was lacking in volatile flavors, and the level of harmful contents were uncertain, therefore it could not be sold as liquor. In this study, the protein, fat, and tannin in the clusters and leaves of sweet sorghum were utilized to increase the content of flavor compounds in the ethanol product through the anaerobic fermentation of *Saccharomyces cerevisiae*. Meanwhile, the silage fermentation method was used to extend the preservation time of the raw materials and to further enhance the flavors of Fen-flavor liquor, with ethyl acetate as the characteristic flavor. The effects of different feedstock groups on ethyl acetate, ethyl lactate, methanol, acetaldehyde, acetal, fusel oil, total acid, and total ester were evaluated by analyzing the chemical composition of different parts of sweet sorghum and determined by gas chromatograph. The effect of different fermentation periods on the volatile flavor of sweet sorghum Baijiu was evaluated. The yield of the characteristic volatile flavor was increased by the extension of the fermentation time. Sweet sorghum Baijiu with a high ester content can be used as a flavoring liquor, blended with liquor with a shorter fermentation period to prepare the finished Fen-flavor Baijiu, conforming to the Chinese national standard for sale.

## 1. Introduction

Baijiu, known as Chinese liquor, is one of the oldest distilled alcoholic beverages in the world, produced by solid-state fermentation and distillation. After thousands of years of historical evolution, three dominating varieties with distinctive flavor profiles have gradually formed, being mild-flavor, strong-flavor, and sauce-flavor, which are represented by Fen-flavor, Luzhou-flavor, and Maotai-flavor, respectively [1,2]. Fen-flavor liquor is characterized by a pure aroma and a mellow sweet scent. The Fen-flavor liquor brewing process can be classified according to starter cultures as the Daqu (large starter cultures) process and the Xiaoqu (small starter cultures) process [3]. Daqu is a saccharification and fermentation stater enriching multiple microorganisms and enzymes air-dried on starchy raw materials. The essential component of Xiaoqu is *Saccharomyces cerevisiae* in a dormant state. Saccharomyces cerevisiae not only produces ethanol, but also produces various volatile flavors with raw materials during fermentation [4,5,6].

Sorghum grain is the most commonly used raw material in Baijiu, especially suitable for Xiaoqu Fen-flavor liquor. Sorghum can be classified into grain sorghum and sweet sorghum according to its usage. Grain sorghum is used for food and liquor, while sweet sorghum is normally utilized for sugar and biofuel ethanol production [7,8,9,10]. Sweet sorghum stalks are rich in fermentable sugars such as sucrose, fructose, and glucose, which can be directly digested by *Saccharomyces cerevisiae* to produce alcohol.

With the development of continuous solid-state fermentation and distillation technology, the advantages of sweet sorghum as an energy crop has become more prominent [11,12,13]. Previous studies have focused on the use of sweet sorghum stalks to produce sugar and ruminant animal feed, but little attention has been paid to the function of sweet sorghum grains as a raw material of liquor [14,15]. The bioethanol production process from sweet sorghum stalks by solid-state fermentation is basically consistent with the Xiaoqu Fen-flavor Baijiu brewing process, but the focuses of the two studies are different. Biofuel researchers highlighted the energy available, while liquor researchers paid more attention to the volatile flavors produced during the fermentation. Baijiu is composed of 98%–99% ethanol and water plus 1%–2% trace organic ingredients. These ingredients are slight in content but abundant in variety, which constitutes the flavor profile of Baijiu.

Most of the fermentable substances in sweet sorghum stalks are sugar, which means that the Baijiu fermented by *Saccharomyces cerevisiae* strains is almost all ethanol but lacking in volatile flavors. Sweet sorghum grains are rich in starch, protein, fat, and tannin, which could affect the flavor compound production [16]. The main components in sweet sorghum leaves are protein, fat, and ash, which could be also utilized to enhance the flavor compounds. Therefore, it is important to investigate the effect of how the sweet sorghum grains and leaves influence the stalk fermentation and flavor production. It is also essential to evaluate whether the whole sweet sorghum bagasse plant can be used as raw material for Baijiu fermentation.

Silage fermentation is a type of solid-state fermentation and is widely applied in feed production. Lactobacillus bacteria are normally used as silage starters for crops to produce amounts of lactic acid through anaerobic respiration, which can inhibit the growth of harmful bacteria and preserve the nutrition of green forage for long periods [17,18]. When *Saccharomyces cerevisiae* is used as silage starter for sugar crops such as sweet sorghum, the ethanol produced can also inhibit the harmful bacteria [19]. Compared with traditional solid-state fermentation, silage fermentation significantly prolonged the fermentation period. Evidently, the prolongation of fermentation time is conducive to the enrichment of various microorganisms on feedstock to produce flavor compounds, but a quantitative evaluation is required.

In this work, the chemical composition of different parts of the whole sweet sorghum plant were analyzed. The effect of sweet sorghum clusters and leaves on stalk fermentation was assessed by flavor compound analysis. Subsequently, the fermentation time was extended to observe the change in volatile flavor contents. The optimal raw material group and silage fermentation period were determined according to the results. It would be helpful to establish a food-saving Baijiu production process with low-cost raw materials.

## 2. Materials and Methods

### 2.1. Materials

#### 2.1.1. Sweet Sorghum

The sweet sorghum cultivar “LiaoTian 1#” was harvested in September 2019 in Inner Mongolia, China. The total growing period was 136 days. Harvested whole crops were stored in the field and transported to the laboratory for about 5 days. The stalks, leaves, and clusters were crushed separately by a pulverizer and stored in a refrigerator at −10 °C before fermentation. The pulverized particles were rod-shaped, and the dimension of most particles was 2 × (20–30) mm (diameter × length).

#### 2.1.2. Yeast Strain

*Saccharomyces cerevisiae* TSH1 deposited in the China General Microbiological Culture Collection Center (Accession number: CGMCC 1949) was used in this study. The TSH1 yeast strain was isolated from the soil sample for the long-term storage of sweet sorghum. We identified that the yeast had significant advantages for use in solid-state fermentation, including a tolerance to temperature differences and a low moisture content [20,21].

### 2.2. Experiment Operation

**Figure 1:**
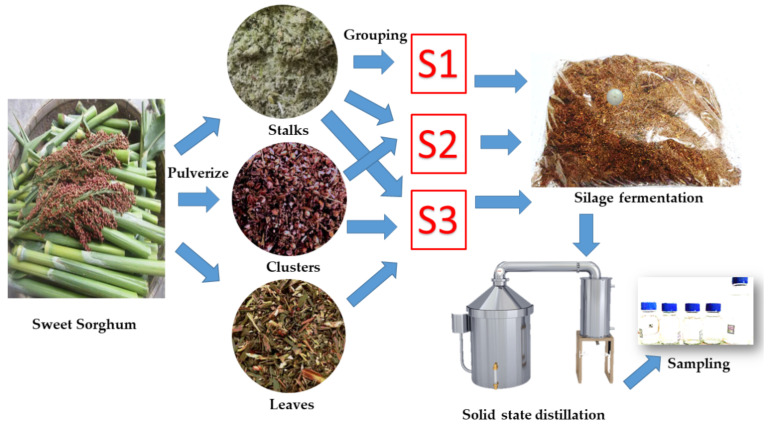
Experiment operation flow diagram.

The operation process of the experiment is shown in Figure 1, including the raw material pulverizing, grouping, silage fermentation, solid-state distillation, and sampling.

#### 2.2.1. Raw Material Grouping

Considering that sweet sorghum plants can be defoliated and threshed during harvest, clusters and leaves can be mixed with stalks separately. The bagasse was manually divided into 3 groups in this study: (1) S1 sweet sorghum stalks; (2) S2 sweet sorghum stalks + clusters; and (3) S3 sweet sorghum stalks + clusters + leaves.

The stalks, clusters, and leaves of sweet sorghum were separated by hand, pulverized, and weighted respectively. The cluster includes sorghum grains, husks, and connecting branches. Before grouping, 0.2 wt% glucoamylase was sprayed on pulverized sorghum clusters to promote the conversion of starch into glucose without pasting. The proportions of different parts of S2 and S3 were determined according to the average wet weight ratio of each part of sweet sorghum measured after pulverizing.

#### 2.2.2. Silage Fermentation

From each divided group, 10 kg of bagasse was disinfected with sulfur dioxide to kill miscellaneous bacteria in the sweet sorghum, which is beneficial to ethanol production and feedstock preservation. Sulfur dioxide can be employed as a wine or liquor preservative and the residual concentration after long-term preservation conforms to Chinese food additive standards [22]. A 100 g/L sulfur dioxide solution was prepared by adding 167 g sodium metabisulfite (AR ≥ 96%, analytical grade, general reagent, Shanghai, China) into 1 kg deionized water and was sprayed on samples based on different storage times. For the preservation periods of 90, 180, and 360 days, the concentrations of sulfur dioxide sprayed were 500 ppm, 1000 ppm, and 2000 ppm, respectively.

TSH1 strains were frozen with glycerol at −80 °C before incubating. Yeast cells were pre-cultured in YPD broth (10 g/L yeast extract, 20 g/L glucose, and 20 g/L peptone, Sangon biotech, China), incubated at 30 °C with an agitation rate of 200 rpm for 12 h. Next, yeast was inoculated into a secondary fermentation medium for a ten-fold expansion, incubated at the same temperature and agitation rate for 6 h. After being washed with sterilized water, the cultivation of yeast was completed when OD_600_ = 10 was measured by a spectrophotometer (UVmini-1240, Shimadzu, Kyoto, Japan), which means about 1 × 10^8^ viable cells per milliliter [22].

Cultured starter was inoculated into the raw material as a proportion of 2 wt%. Next, samples were sealed in fermentation bags. Each group of treated sample was packed into three bags, as parallel samples for fermentation, distillation, sampling, and flavor compounds testing. The bags were made of PE+PA composite membrane material and equipped with a single-way vent valve for releasing carbon dioxide produced in anaerobic fermentation to avoid the bags breaking. All bags were stored outdoors at ambient temperature (0–30) °C for a specified period.

#### 2.2.3. Baijiu Distillation

After the fermentation, the bagasse was loaded into a distiller for solid-state distillation. The distiller was made of stainless steel, with an inner diameter of 320 mm. It was equipped with a conical head on the top and a steam distributor on the bottom. The loading chamber and the steam chamber were separated by an 80-mesh screen sintered plate. The distiller outlet was connected to a condenser with a heat transferring area of 1 m^2^.

Sample bottles were used to collect liquor at the condenser outlet. Samples of 250 mL were continuously collected 4 times, and 1000 mL samples were collected once. The ethanol content and acidity were measured in each sample. Next, liquor samples were blended in proportion to the alcohol degree of 50 *v/v*% for further volatile flavor analysis.

### 2.3. Analytical Methods

The moisture content in the sweet sorghum was determined by drying it at 105 °C for 12 h (GB/T6435). The pulverized sweet sorghum characterized by crude protein (GB/T6432), crude fat (GB/T6433), crude fiber (GB/T6434), and ash (GB/T6438) contents were determined by Chinese standard procedure [23,24,25,26,27]. Tannin content and total starch content were determined by a dual-wavelength spectrophotometric method [28]. High performance liquid chromatograph (LC-20AD, Shimadzu, Kyoto, Japan) equipped with an ion-exchange column (Aminex HPX-87H, Bio-Rad, CA, USA) and a differential refractive index detector was used to determine the ethanol and fermentable sugar concentration. The column temperature was 40 °C, and 0.05 mol/L sulfuric acid was prepared as a mobile phase at a flow rate of 0.6 mL/min [29].

Volatile flavors were analyzed by a gas chromatograph (Clarus 600, PerkinElemer, MA, USA) equipped with a flame ionization detector (GC-FID). The carrier gas was helium with a purity of 99.9995%. The CP-Wax 57CB column (50 m × 0.25 mm I.D. × 0.20 µm df) was purchased from Agilent Technologies (Wilmington, DE, USA). The gas chromatograph was equipped with a split-splitless injector which was held at 240 °C. Injections were performed in a split mode with a split ratio of 1:20. The column temperature was held at 40 °C for 3 min and increased to 80 °C at 4 °C/min. Subsequently, the column temperature was increased to 130 °C at 9 °C/min followed by an increase to 205 °C at 15 °C/min for 15 min. The hydrogen and air flow rates on the flame ionization detection were 45 mL/min and 350 mL/min, respectively. [30,31,32]. Each expectant flavor chemical was used to prepare the standard solutions with different gradient concentrations. The standard curves for each compound was drawn based on GC detection data. The correlation coefficient of the standard curve for each compound is above 0.999. An internal standard method was used. The internal standard was methyl-2-butanol, n-butyl acetate, and 2-ethyl butyric acid. The samples were injected with the inner standard solution without any extraction. The sample volume was 1 µL.

Statistical significance was performed using one-way analysis of variance (ANOVA) and multiple comparison using Tukey’s HSD with SPSS version 19.0 software (IBM, New York, USA). Data were considered to be statistically significant at *p* < 0.05. The flavor compound differences of four sampling groups were analyzed by SPSS software.

## 3. Results and Discussion

### 3.1. Chemical Compositions of Different Sweet Sorghum Parts

Table 1 shows the weight percentage of different parts of sweet sorghum. The stalk accounts for more than 85% of the weight of the whole plant. Clusters weigh slightly more than leaves. The weight proportion of the stalk in group S1 to S3 was 100%, 91.8%, and 86.3%, respectively.

Sweet sorghum stalk contains the most moisture, while the leaf contains less water than the stalk. The large surface area of the leaves results in a portion of the water being evaporated during field storage. The atmospheric drying of the leaves reduces the risk of mildew. The medullary structure beneath the epidermis causes most of the moisture to be maintained in the stalks. The water in the grains can also be preserved in the husks. Thus, there was little moisture loss in the stalks and clusters during field storage and transportation. After grouping, the moisture in each fermentation group was within the optimal water activity range for TSH1 yeast growth [33]. There was no mobile water in the silage bags, which are suitable for solid-state fermentation.

The chemical compositions of each part of sweet sorghum under dry base conditions are shown in Table 2. Values of each composition are significantly different at *p* < 0.05.

Sweet sorghum stalk is rich in fermentable sugars such as fructose, sucrose, and glucose, which can be directly digested by yeast to produce ethanol and is the most important carbon source in Baijiu making. A large amount of fermentable sugars in all three fermentation groups are beneficial for yeast to produce an ethanol atmosphere in the silage bags, which limits the growth of other bacteria and fungi in the early stage of fermentation. According to our previous research results, the average glucose metabolism rate of the TSH1 strain under the conditions of initial glucose concentration of 60 g/kg, standing solid state, and 20 °C is about 2.5 × 10^−4^ min^−1^ [33]. After 7 days of storage, 90% of fermentable sugars can be converted into ethanol.

Starch is mainly contained in sweet sorghum grains, the majority of which is amylopectin [34]. By pretreating pulverized clusters with glucoamylase, the starch can be converted to glucose without pasting. Glucoamylase can hydrolyze a α-1,4 glycosidic bond of raw starch at the non-reducing end, and slowly hydrolyze a α-1,6 glycosidic bond to glucose. The hydrolysis rate of raw starch of glucoamylase is related to whether it can be adsorbed by native starch granules and the adsorption intensity. Raw starch has a certain adsorption capacity and affinity capacity for glucoamylase, which makes glucoamylase more accessible to starch granules and catalyzes the hydrolysis of starch to glucose [35]. In the process of silage fermentation, starch in grains can be hydrolyzed to glucose synchronously with yeast growth, thereby increasing the ethanol concentration of feedstock.

Most proteins come from sweet sorghum leaves and grains. During fermentation, protein can provide nitrogen for *Saccharomyces cerevisiae* growth and metabolism to prompt the production of higher alcohols, amino acids, and esters. However, an excessive crude protein content would produce off-flavor in liquor and make the feedstock contaminated by sundry bacteria.

Crude fat can be converted to fatty acid as precursors of esters via lipolysis. Corn is often used to increase the fat content of the raw material in traditional Baijiu making [36]. However, an increased fat content will increase the calorific value of the fermentation, so that the rapid growth and reproduction of sundry bacteria will affect the flavors of liquor.

Therefore, the content of crude fat and crude protein in the raw material should be controlled within a reasonable range. As shown in Table 2, the stalks had a much lower level of fat and protein. The addition of leaves and clusters increased the amount of crude fat and crude protein, which was conducive to the production of flavors. However, it is not known how the additional fat and protein content will affect the liquor quality until the volatile flavors have been tested.

Tannin was found almost exclusively in the cluster of sweet sorghum. In fact, a higher content of tannin was detected in husks than grains. Trace tannin can produce phenolic compounds such as syringic acid to increase the aromatic flavor of liquor. However, when the content of tannin exceeds 1.94 wt%, the liquor will have a bitter taste and the microbial metabolism would be inhibited [36]. The tannin content was much lower than the reported value in all three groups in this study, which could be beneficial for flavor production.

The crude fiber in sweet sorghum could not be directly used in the metabolism of yeast, but it provided a solid-state substrate for silage fermentation. Without mobile water, the reproduction of miscellaneous bacteria was inhibited, which is the basis for the long-term preservation of fermented bagasse. Fermentation products were also kept in the framework of lignocellulose to reduce the loss of volatile flavor compounds during the silage period.

Ash is one of the essential components of sweet sorghum leaves. The ash contains trace elements such as silicon, potassium, sodium, and iron, which are beneficial for the growth of microorganisms. These trace elements have little effect on the flavor characteristics of Baijiu, but could affect the quality of vinasse feedstuffs.

As stated above, almost all fermentable components in group S1 are sugar, which is the same as the raw material for fuel ethanol production. It can be inferred that the volatile flavor content in distilled liquor from S1 will be at a low level. The components of starch, protein, and tannin were increased when sweet sorghum clusters were added in S2. The leaves added in S3 further increased the proportion of protein and fat. How these components improved the production of various flavor compounds will be discussed in the following section.

### 3.2. Volatile Flavors of Different Fermentation Groups

#### 3.2.1. Spectra of Standard Sample

**Figure 2:**
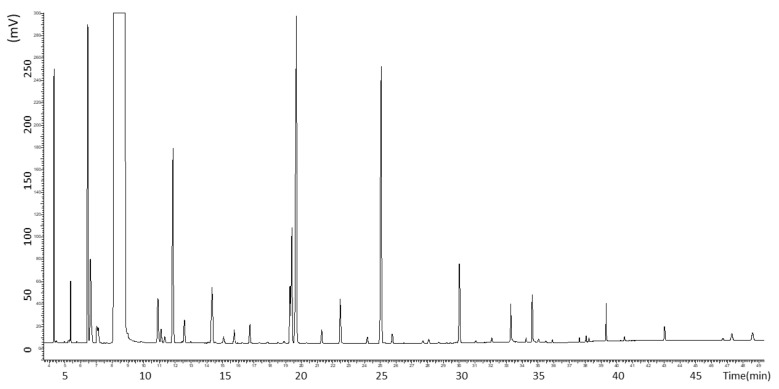
Standard sample spectra of volatile compounds.

The standard sample spectra of 48 compounds of liquor is shown in Figure 2. Each component can be completely separated under the chromatographic conditions, which satisfied the requirements of this study. They are classified as alcohols, aldehydes, ketones, and esters. This article will highlight important flavor substances for discussion.

#### 3.2.2. Flavor Compounds in Sweet Sorghum Baijiu

According to the chromatographic conditions, the main components in sweet sorghum Baijiu were acetaldehyde, methanol, ethyl acetate, n-propanol, isobutanol, isopentanol, acetal, and ethyl lactate, which were common compounds in Fen-flavor liquor besides ethanol and water. The Fen-flavor Baijiu is characterized by a harmonious complex aroma dominated by ethyl acetate, pure and long fragrance, slightly sweet in the mouth, strong stimulation, and a slightly bitter taste [37]. The flavors can be divided into three types, namely, esters, higher alcohols, and carbonyl compounds.

Esters are generated by an acid-alcohol reaction, among which is ethyl acetate, which is the characteristic compound of Fen-flavor liquor. The ethyl lactate content is second to ethyl acetate, and its flavor is not as strong as ethyl acetate. Other esters, found in trace content, also contribute to the flavor of Baijiu. For example, diethyl succinate has a faint fruit odor, and when interacting with β-phenylethanol, can produce a luscious aroma [2]. Various esters, including ethyl palmitate, ethyl oleate, ethyl linoleate, and ethyl tetradecanoate, together with ethyl acetate and ethyl lactate, were evaluated by the total ester content in this study.

Organic acids in liquor are precursors of esters and more than 90% of them are acetic acid and lactic acid [31]. Appropriate organic acids are helpful in enhancing the aftertaste of liquor. During fermentation, preservation, and distillation, a part of organic acid reacts with ethanol. The remaining organic acids were evaluated as the total acidity in this study.

Higher alcohols contribute to the taste and aroma of Fen-flavor liquor and are also precursors of esters [31]. The higher alcohols in liquor are mainly n-propanol, isopentanol, and isobutanol. The taste of higher alcohols is fairly bitter and astringent, forming the clear and refreshing characteristics of the Fen-flavor liquor. Other higher alcohols, such as β-phenylethanol and 2, 3-butanediol, also had effects on the liquor flavor. Hence, the content of total higher alcohols was evaluated as fusel oil in this study.

Carbonyl compounds contribute the stimulating and spicy taste, as well as promoting and enhancing the aroma of the liquor body. Acetaldehyde and acetal account for more than 95% of all carbonyl compounds. Acetaldehyde could stimulate gustatory cells to pain, and are a source of pungency but harmful to human health. Acetal emits a gentle and refreshing aroma, which could neutralize the stimulation of acetaldehyde to a certain extent. The preferred ratio of acetaldehyde to acetal is 1 to (1.2–2) as reported [38].

Methanol produced during fermentation is harmful to human health. Its concentration should be strictly limited. Methanol has a mild alcohol odor, but it has an inhibitory effect on the central nervous system, especially on the retinal nervous system. Methanol is oxidized to formaldehyde and formic acid in the body, making it even more toxic. Therefore, standards have strict restrictions on methanol content in liquor [39].

#### 3.2.3. Effects of Sweet Sorghum Leaves and Clusters on Volatile Flavors

To analyze the contribution of different parts of sweet sorghum to the liquor flavors, a 50 *v/v*% commercial Fen-flavor Baijiu fermented from sorghum grain was used as the control group of S0.

**Figure 3:**
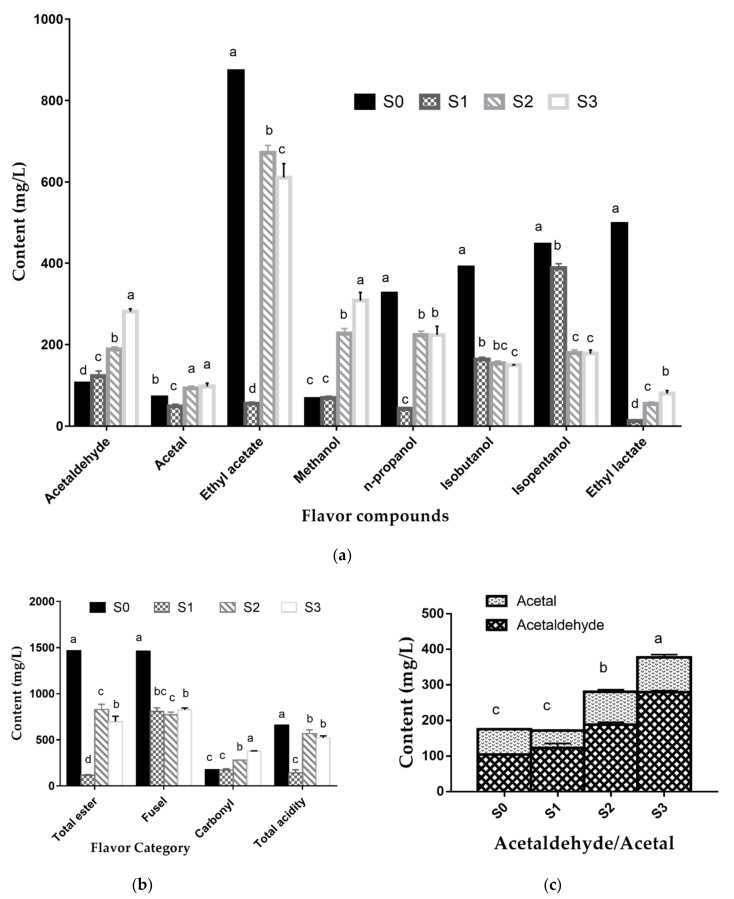
Content of flavor compounds in each treated group. Different letters (a, b, c, d) on the bars indicate significant differences (*p* < 0.05) between different samples. (**a**) Comparison of characteristic volatile flavors; (**b**) comparison of flavor categories; (**c**) comparison of the ratio of acetaldehyde to acetal.

The results of flavor compounds in four groups are presented in Figure 3. Compared with the control group, there were significant (*p* < 0.05) differences in the content of volatile flavors in all three treated groups, except the methanol content in S1. There were no significant differences in the content of acetal, n-propanol, isobutanol, isopentanol, and total acidity between S2 and S3, indicating that the addition of sorghum leaves and clusters had almost the same effect on acetal, organic acid, and higher alcohols production. S1, S2, and S3 had no significant differences in total fusel oil content, which was much lower than in S0, as displayed in Figure 3b.

The metabolic mechanisms of fusel oil include anabolic metabolism and catabolic metabolism. Their carbon sources are glucose and amino acids, respectively. Previous studies have shown that 75% of the low-chain higher alcohols in liquor, such as isopentanol, isobutanol, and reactive pentanol, are produced by anabolic metabolism, and 25% of them come from catabolic metabolism [40]. Proteins in sorghum clusters and leaves changed the composition of carbon sources in feedstock and the proportion of various higher alcohols in total fusel oil. However, glucose anabolic metabolism by yeast was still the main source of higher alcohols, so the total amount of fusel oil changed little. Excessive fusel oil content is the reason for discomfort after drinking, so it should be limited during fermentation [41,42]. A low fusel oil content can be seen as one of the advantages of sweet sorghum Baijiu.

What stands out in Figure 3a is the growth of ethyl acetate content with the addition of sweet sorghum clusters. After adding sorghum clusters to S1, the content of ethyl acetate increased by nearly 12 times, indicating that fermentation of clusters is the main source of ethyl acetate production. The content of ethyl acetate decreased slightly as sorghum leaves were added into the S2 group. It can be concluded that sorghum leaves also had an effect on ester production, but only about one-tenth of the clusters. When sweet sorghum stalks were fermented alone, almost all the products of anaerobic digestion from fermentable sugars were ethanol. Sorghum clusters enriched the metabolic pathway of yeast by introducing protein, fat, tannin, and starch into the raw materials. An acid-alcohol reaction enabled the ethyl acetate content to increase during preservation. As the characteristic volatile flavor of Fen-flavor liquor, the content of ethyl acetate in S2 is close to the level in commercial liquor.

As displayed in Figure 3a, the addition of the sorghum clusters and leaves did not significantly increase the content of ethyl lactate, the secondary characteristic flavor compound of Fen-flavor, which was significantly lower than in the control group. The content of ethyl lactate is determined by the amount of lactic acid produced during fermentation, which is primarily limited by the fermentation method and strains used. The reason for the high content of ethyl lactate in the control group is that a variety of microbial communities, including Rhizopus, lactic acid bacteria, and acetic acid bacteria, besides *Saccharomyces cerevisiae*, fermented on starch to produce lactic acid [43]. In this study, in order to form an ethanol anaerobic atmosphere in the initial stage of silage fermentation, only *Saccharomyces cerevisiae* was used as starter. The ethanol atmosphere inhibited the growth of other strains, including lactic acid bacteria, to achieve long-term preservation. This also indicated that *S. cerevisiae* has almost no metabolic pathway to produce lactic acid from sugar.

However, the effect of ethyl lactate on Fen-flavor was not obvious due to its diluted aroma and high boiling point. Several studies have revealed that ethyl lactate could inhibit other flavor compounds, so the content of ethyl lactate was often controlled in Baijiu [44,45]. The low content of ethyl lactate in sweet sorghum Baijiu is conducive to highlighting the characteristic flavor of ethyl acetate. Other higher esters such as ethyl butyrate, ethyl caproate, ethyl palmitate, ethyl oleate, ethyl linoleate, ethyl tetradecanoate, etc., are not the main flavor compounds of Fen-flavor liquor. The contents of these esters in the control group and treated groups were similar based on GC-FID analysis results.

S1 and S0 had the lowest level of methanol and the difference was not significant (*p* < 0.05). When clusters and leaves were mixed, the methanol level increased to the highest in S3, but it was still lower than the concentration of 600 mg/L required in the Chinese national standards [39].

Besides acetic acid and lactic acid, most of organic acids above three carbons have a higher boiling point than water and are difficult to volatilize during distillation. The proportion of organic acids participating in an acid-alcohol reaction and the unreacted organic acids in each group was similar. Because of the low content of lactic acid, the total acidity of sweet sorghum liquor has a high correlation with the change of ethyl acetate content, as shown in Figure 3b.

In terms of carbonyl compounds, as shown in Figure 3b,c, the levels in S2 and S3 were higher than those in S0 and S1. The fermentation of leaves in S3 especially increased the content of acetaldehyde and the spicy taste of the liquor. S2 was closer to the optimum ratio of acetaldehyde and acetal mentioned above [38]. It was worth noting that both acetaldehyde and methanol were harmful components in Baijiu, so the addition of leaves will affect the quality of sweet sorghum liquor to some extent.

### 3.3. Effect of Silage Fermentation Period on Volatile Flavors

**Figure 4:**
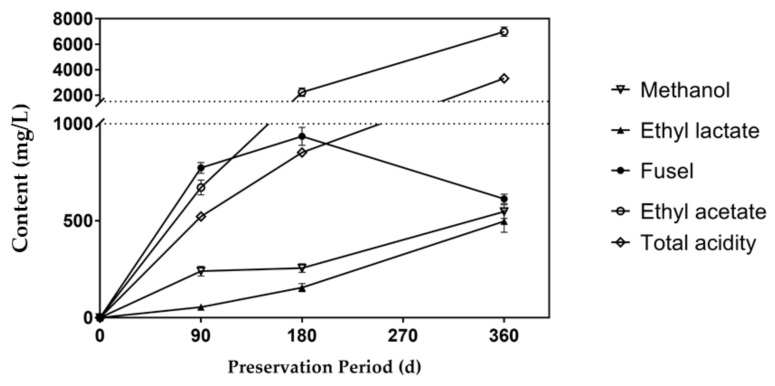
Effect of preservation period on volatile flavors.

Xiaoqu Fen-flavor liquor is characterized by a rapid fermentation process with a fermentation period of 5 to 7 days. Sweet sorghum leaves and stalks have a high moisture content. Unlike grains that can be preserved throughout the year, sweet sorghum can be stored under ventilation for less than 1 month with a high sugar loss. In order to meet the demand of annual production, the silage fermentation period of sweet sorghum bagasse was expected to be 90 to 360 days. Based on the discussion above, the S2 group had the best liquor quality. Therefore, the S2 samples were further preserved in silage bags for 180 days and 360 days, respectively. The liquor was distilled and analyzed with the same methods. The results were compared with those in the 90-day storage period, as shown in Figure 4.

The content of ethyl acetate increased significantly as the fermentation time was extended. This is because the organic acids in liquor are dependent on raw materials and microbial metabolism. When the acidity in the preservation environment reduced, the transformation pathway of residual sugar changed. Acetaldehyde is an intermediate product of anaerobic fermentation, usually affected by a hydrogen acceptor. When the hydrogen ion concentration decreased, the disproportionation reaction of acetaldehyde occurred, leading to mutual oxidation and the reduction of two acetaldehyde molecules to generate one ethanol molecule and one acetic acid molecule. In addition, the initial completely sealed environment was gradually destroyed with the extension of the fermentation period. The carbon dioxide and a small fraction of ethanol vapor were exchanged with ambient air through the breathing valve. Acetic acid and lactic acid bacteria are widespread in nature and might enter the fermentation bag with air. Changes in the fermentation atmosphere could also activate the bacteria that were originally dormant in the plants to produce organic acids using residual sugar in feedstock. Acetic acid and lactic acid were gradually esterified with ethanol, increasing the ethyl acetate and ethyl lactate contents in the sweet sorghum Baijiu, as described in Figure 4.

Specifically, the ethyl lactate content in sweet sorghum Baijiu after 360 days of fermentation was close to the level of commercial Fen-flavor Baijiu. Ethyl acetate has a high volatility, and the aroma is not lasting. Therefore, the specification of Fen-flavor liquor recommends the ethyl acetate content less than 2600 mg/L [37]. The increasing trend of total acidity is similar as that of the ester content. With the extension of the fermentation time, the methanol content also increased, but still less than the standard value. After 360 days of fermentation, the content of fusel oil decreased further. The comparison of flavor compounds at different fermentation times with the specification of Fen-flavor liquor is given in Table 3. The ethyl acetate content of liquor fermented for 360 days was higher than the standard value, but it was suitable for flavoring liquor, mixed with the liquor with a shorter fermentation period to obtain a better flavor. The finished mixed liquor can be packaged for sale.

## 4. Conclusions

The present study set out to find a new method for producing Fen-flavor Baijiu by silage fermentation with the sweet sorghum whole plant as raw material. Fermentable sugars in the sweet sorghum stalk were fully utilized to produce ethanol. Fat and protein in the clusters and leaves were used to increase the volatile flavors of liquor making.

The findings of this study suggest that the addition of sweet sorghum clusters and leaves improved the flavor compounds in fermented stalks liquor. The treated group S2 composed of stalks and clusters produced more ethyl acetate and total esters, less methanol, and a more suitable acetaldehyde to acetal ratio. Sorghum leaves can partially replace clusters as the source of volatile flavor, but can increase the content of harmful compounds such as methanol and acetaldehyde. Therefore, the group of clusters and stalks was regarded as the most suitable feedstock for sweet sorghum Baijiu making. It is also feasible to use the pulverized whole plant of sweet sorghum without automatic leaf stripping at harvest.

Extending the fermentation period can further increase the ester content in liquor to reach a high level. As Fen-flavor Baijiu quests ethyl acetate aroma, the flavor and overall quality of Baijiu are improved during the fermentation period extension. The fermented liquor conforms to the Chinese national standard for Fen-flavor Baijiu and the flavor profile of the liquor is consistent with the Fen-flavor.

This cost-effective technology has advantages such as a low raw material cost and food saving. The approach will be proved especially applicable for distributed cattle farms with sweet sorghum as feed. Silage fermentation can ensure the supply of cattle feed throughout the year together with the production of high-quality Fen-flavor Baijiu. This will greatly improve the economic value of sweet sorghum. Therefore, this work provides a new Baijiu production process with excellent economic benefits and an outstanding application prospect.

## Figures and Tables

**Table 1:** Percentage of weight and moisture of different sweet sorghum parts and each experimental group (wt% wet basis).

Items	Clusters	Leaves	Stalk	S1	S2	S3
Weight	7.75	5.93	86.32	Stalk	Clusters + Stalk	Clusters + Leaves + Stalk
Moisture ^1^	25.6 ± 0.15	46.9 ± 0.13	71.1 ± 0.16	71.1 ± 0.16	67.4 ± 0.16	66.1 ± 0.15

^1^ Results were given as averages of 3 replicates ± standard deviation.

**Table 2:** Chemical compositions of different parts of sweet sorghum (wt% dry basis).

Parts	Fermentable Sugars ^1^	Total Starch ^1^	Crude Protein ^1^	Crude Fat ^1^	Crude Fiber ^1^	Ash ^1^	Tannin ^1^
Clusters	2.67 ± 0.03	34.33 ± 0.05	10.29 ± 0.03	2.63 ± 0.01	13.97 ± 0.03	4.35 ± 0.04	1.72 ± 0.02
Leaves	UD ^2^	3.24 ± 0.02	11.37 ± 0.02	2.53 ± 0.03	24.85 ± 0.37	9.04 ± 0.02	0.09 ± 0.00
Stalk	41.88 ± 0.21	10.00 ± 0.12	4.49 ± 0.03	0.98 ± 0.05	29.25 ± 0.24	5.27 ± 0.01	0.03 ± 0.00

^1^ Results were given as averages of 3 replicates ± standard deviation. ^2^ UD means undetected.

**Table 3:** Comparison of liquor flavors between different fermentation period Baijiu and standard values of Fen-flavor Baijiu.

Item	Standard Values (mg/L)	Literature ^2^	90 Days ^1^ (mg/L)	180 Days ^1^ (mg/L)	360 Days ^1^ (mg/L)
**Methanol**	<600	[39]	236 ± 21.7	258 ± 9.4	551 ± 29.2
**Total acidity**	>400	[37]	521 ± 21.1	852 ±16.1	3319 ± 145.7
**Total ester**	>1000	[37]	826 ± 13.1	2287 ± 142.4	7619 ± 146.5
**Ethyl acetate**	600–2600	[37]	674 ± 31.4	2136 ± 165.1	7051 ± 252.7

^1^ Results were given as averages of 3 replicates ± standard deviation. ^2^ According to Chinese standards nomenclature, GB means mandatory standards and GB/T means recommended standards.

## Data Availability

The data presented in this study are available on request from the corresponding author.
